# Gene expression profiles of breast cancer metastasis according to organ site

**DOI:** 10.1002/1878-0261.13021

**Published:** 2021-06-23

**Authors:** Fara Brasó‐Maristany, Laia Paré, Nuria Chic, Olga Martínez‐Sáez, Tomás Pascual, Meritxell Mallafré‐Larrosa, Francesco Schettini, Blanca González‐Farré, Esther Sanfeliu, Débora Martínez, Patricia Galván, Esther Barnadas, Belinda Salinas, Pablo Tolosa, Eva Ciruelos, Esther Carcelero, Cecilia Guillén, Barbara Adamo, Reinaldo Moreno, Maria Vidal, Montserrat Muñoz, Aleix Prat

**Affiliations:** ^1^ Translational Genomics and Targeted Therapies in Solid Tumors August Pi i Sunyer Biomedical Research Institute (IDIBAPS) Barcelona Spain; ^2^ Department of Medical Oncology Hospital Clínic of Barcelona Spain; ^3^ SOLTI Cooperative Group Barcelona Spain; ^4^ Department of Pathology Hospital Clínic de Barcelona Spain; ^5^ Department of Clinical Oncology University Hospital 12 de Octubre Madrid Spain; ^6^ Department of Pharmacy Hospital Clínic of Barcelona Spain; ^7^ Department of Oncology IOB Institute of Oncology Quironsalud Group Barcelona Spain; ^8^ Department of Medicine University of Barcelona Spain

**Keywords:** breast cancer, gene expression profiling, HER2‐low, metastatic sites, PAM50

## Abstract

In advanced breast cancer, biomarker identification and patient selection using a metastatic tumor biopsy is becoming more necessary. However, the biology of metastasis according to the organ site is largely unknown. Here, we evaluated the expression of 771 genes in 184 metastatic samples across 11 organs, including liver, lung, brain, and bone, and made the following observations. First, all PAM50 molecular intrinsic subtypes were represented across organs and within immunohistochemistry‐based groups. Second, HER2‐low disease was identified across all organ sites, including bone, and HER2 expression significantly correlated with *ERBB2* expression. Third, the majority of expression variation was explained by intrinsic subtype and not organ of metastasis. Fourth, subtypes and individual subtype‐related genes/signatures were significantly associated with overall survival. Fifth, we identified 74 genes whose expression was organ‐specific and subtype‐independent. Finally, immune profiles were found more expressed in lung compared to brain or liver metastasis. Our results suggest that relevant tumor biology can be captured in metastatic tissues across a variety of organ sites; however, unique biological features according to organ site were also identified and future studies should explore their implications in diagnostic and therapeutic interventions.

## Graphical abstract

We evaluated gene expression in 184 metastatic breast cancer samples across 11 organs. All PAM50 subtypes were represented across metastatic sites and associated with survival. HER2‐low disease was identified across sites. Immune gene expression was higher in lung than brain or liver metastasis. Relevant tumor biology can be captured in metastatic tissue, while each site shows unique biological features.

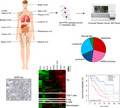

**Abbreviations**
ASCO/CAP: American Society of Clinical Oncologists/College of American Pathologists
Cor: correlation
DAVID: database for annotation, visualization and integrated discovery
ER: estrogen receptor
FDR: false discovery rate
FFPE: formalin‐fixed paraffin‐embedded
GO: gene ontology
HER2+: HER2‐positive
HR: hormone receptor
HR+: hormone receptor‐positive
IHC: immunohistochemistry
ISH: *in situ* hybridization
KEGG: Kyoto Encyclopedia of Genes and Genomes
N/A: not available
OS: overall survival
*P*
: p‐value
PCA: principal component analysis
ROR: risk of recurrence
SAM: significance analysis of microarrays
T-DXd: trastuzumab deruxtecan
TNBC: triple-negative breast cancer

## 1 Introduction

Advanced or metastatic breast cancer affects multiple organs and is a main cause of cancer death [1]. Common metastatic sites include bone, liver, lung, brain, lymph node, pleura, and skin [1, 2, 3, 4, 5, 6]. Interestingly, the different breast cancer intrinsic subtypes (i.e., luminal A and B, HER2‐enriched, and basal‐like) have distinct preferred metastatic sites [7], and both the tumor cell and the metastatic microenvironment might contribute to this organ specificity [8]. To date, however, the biology of breast cancer metastasis according to organ site remains largely unknown.

In advanced breast cancer, biomarker identification and patient selection using a biopsy from a metastatic lesion is becoming a clinical need. On one hand, it confirms the breast origin of the disease. On the other hand, it allows the identification of predictive biomarkers such as *PIK3CA* mutations or the expression of PD‐L1, HER2, and hormone receptors (HR). Although tumor tissue of primary disease obtained years before remains of value to identify these biomarkers when available [9, 10], significant biological differences exist between primary and metastatic disease [11]. For instance, loss of estrogen receptor (ER) has been reported in about 20% of cases [12], while 3–10% discordance of HER2 gene amplification exists in primary versus metastatic tissue [13]. Moreover, advanced disease is enriched with new genetic alterations such as *ESR1* mutations or the APOBEC genetic signature [14] and with phenotypic changes such as the acquisition of the HER2‐enriched subtype in HR‐positive (HR+)/HER2‐negative disease [11]. Importantly, many of these biological alterations during metastatic disease might lead to resistance and treatment failure [15, 16, 17, 18]. In this direction, clinical trials with novel agents are mandating a metastatic tumor biopsy to select patients based on their tumor’s genomic profile.

One critical question that patients, clinicians, and researchers face is which metastatic lesion is better to biopsy or analyze. In certain cases, the most accessible metastatic lesion is chosen. In other circumstances, different options such as liquid biopsies may be available. A better understanding of the molecular profiles of the different metastatic sites might be of value. For example, PD‐L1 expression in immune cells in triple‐negative breast cancer (TNBC) is not recommended in liver biopsies due to the general lack of immune cells in this organ [19, 20]. Another example is determination of HER2 in bone metastasis, which is generally not recommended for technical reasons due to the decalcification procedures. To improve our understanding of the biology of breast cancer metastasis according to the organ site, we performed a phenotypic and molecular characterization of HER2 and 771 genes in 184 metastatic samples across 11 organs, including liver, lung, brain, and bone.

## 2 Materials and methods

### 2.1 Study population

This retrospective and exploratory study included 184 metastatic tumor samples from 176 patients over the age of 18 years with a histologic diagnosis of metastatic breast cancer detected at the time of diagnosis, at first relapse or after disease progression. Tissues were collected from Hospital Clinic of Barcelona (*n* = 161) and Hospital Universitario 12 de Octubre (*n* = 23) in Madrid between years 2000 and 2019. To be included, patients were required to have a formalin‐fixed paraffin‐embedded (FFPE) tissue sample from a locoregional or a distant metastatic lesion. Primary tumor biopsies were allowed if the biopsy was obtained in the context of *de novo* metastatic disease (*n* = 7). Core biopsies were performed according to the routine clinical practice, and HR and HER2 receptor statuses were determined locally in the metastatic biopsy according to the American Society of Clinical Oncologists (ASCO)/College of American Pathologists (CAP) guidelines [21, 22]. HER2 expression status (positive or negative) assessed by immunohistochemistry (IHC) was available for 163 tumor samples (88.6%), while HER2 detailed expression (HER2‐0, HER2 1+ or HER2 2+, and HER2 3+) was available for 148 tumor samples (80.4%). HER2 *in situ* hybridization (ISH) was performed in HER2 2+ tumor samples. HER2‐low tumors were defined when HER2 was determined as HER2 1+ or HER2 2+ and ISH‐negative was identified. HR status (positive or negative) assessed by IHC was available for 158 tumor samples (85.9%), while detailed % of ER expression was available for 148 tumor samples (80.4%). Moreover, we included 186 FFPE tumor samples from patients with early‐stage breast cancer from Hospital Clinic of Barcelona representative of all PAM50 subtypes. The hospital institutional ethics committee approved the study in accordance with the principles of Good Clinical Practice, the Declaration of Helsinki, and other applicable local regulations. Written informed consent was obtained from all patients before enrollment. Patient data were obtained from the database of medical records (SAP Logon 730) and Historia Clinica Compartida (HC3). The medical records were retrospectively reviewed to obtain the clinical data analyzed in the study.

Finally, a publicly available dataset of 390 primary tumors with types of metastatic spread and microarray data was interrogated [23].

### 2.2 Gene expression analysis

RNA was extracted using the High Pure FFPET RNA isolation kit (Roche, Indianapolis, IN, USA) following the manufacturer’s protocol. One to five 10 μm FFPE slides depending on tumor cellularity were used for each tumor sample, and macrodissection was performed, when needed, to avoid normal tissue contamination. A minimum of 100 ng of total RNA was analyzed at the nCounter platform (NanoString Technologies, Seattle, WA, USA) using the Breast Cancer 360 Panel, which measures the expression of 771 breast cancer‐related genes and 5 housekeeping genes (*ACTB, MRPL19, PSMC4, RPLP0,* and *SF3A1*) [24]. Expression counts were then normalized using custom scripts in r 3.6.3.

### 2.3 PAM50 molecular subtypes and gene signatures

All tumors were assigned to an intrinsic molecular subtype of breast cancer (luminal A, luminal B, HER2‐enriched, basal‐like, and normal‐like) using the previously reported PAM50 subtype predictor [25]. For each sample, we calculated scores for 9 signatures including the 5 PAM50 signatures (luminal A, luminal B, HER2‐enriched, basal‐like, and normal‐like) [11], the proliferation signature [26], two risk of recurrence (ROR) signatures at 10 years: ROR score based on subtype (ROR‐S) and based on subtype and proliferation (ROR‐P) as described previously [26], and the previously reported PAM50MET signature, which is based on 17 variables [27]. Gene expression data will be deposited in the Gene Expression Omnibus under the accession number GSE175692.

### 2.4 Statistical analysis

Chi‐square tests were performed to determine the differences in the distribution of variables. Data were subjected to unsupervised hierarchical clustering and principal component analysis (PCA) to identify patterns of expression and to clean the dataset from outliers, 3 out of 184 samples were excluded. Unpaired and multiclass significance analysis of microarrays (SAM) [28], using false discovery rate (FDR), was used to identify differential gene expression across metastatic sites (*n* = 181). Due to the low number of metastasis from ovary (*n* = 4), muscle (*n* = 2), and peritoneum sites (*n* = 2), these samples were excluded from the multiclass SAM analyses. Logistic regressions were used to identify organ‐specific genes.

Overall survival (OS) was defined as the period of time of first diagnosis of metastatic disease to death or last follow‐up. Censoring was done at 120 months. Estimates of survival were from the Kaplan–Meier curves and tests of differences by the log‐rank test. Univariate and multivariable Cox models were used to test the prognostic significance of each variable. The Bonferroni correction method was used to control the family‐wise error rate in case of multiple comparisons [29]. All differences were considered significant at *P*‐value < 0.05. All statistical computations were carried out in r 3.6.3 (http://cran.r‐project.org).

### 2.5 Functional and pathway enrichment analyses

Gene ontology (GO) annotation analysis and Kyoto Encyclopedia of Genes and Genomes (KEGG) pathway enrichment analysis were analyzed by the Database for Annotation, Visualization and Integrated Discovery (DAVID, http://david.abcc.ncifcrf.gov/) online tool [30]. The list of 771 available genes was used as the background or reference gene list. A *P* < 0.05 was considered statistically significant.

## 3 Results

### 3.1 Patients and samples characteristics

**Fig. 1:**
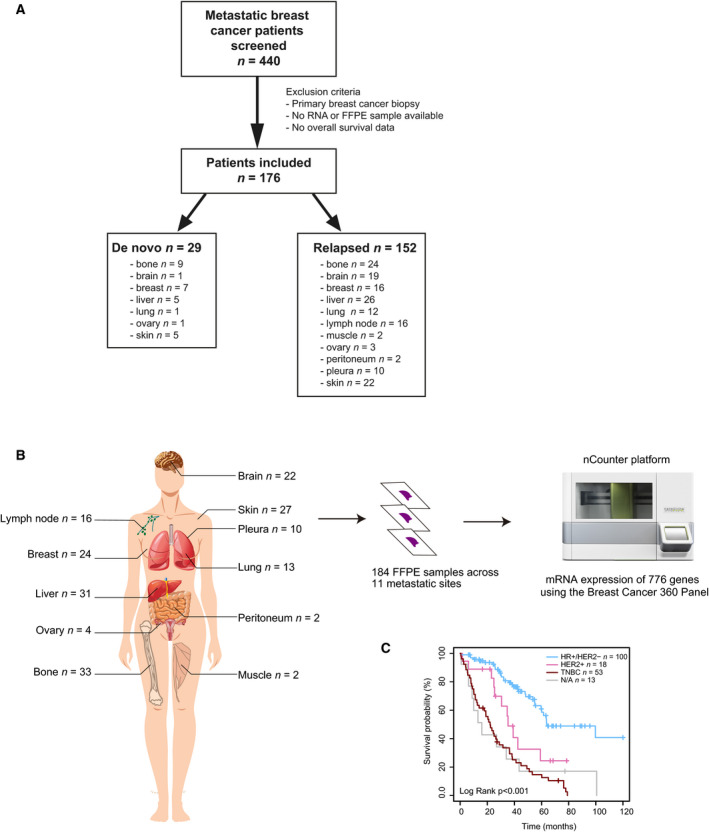
Sample characteristics. (A) Consort diagram reflecting the number of tumor samples evaluated in the study. (B) RNA extracted from 184 FFPE tumor samples obtained from 11 different metastatic sites was analyzed at the nCounter platform using the Breast Cancer 360 Panel. (C) Kaplan–Meier curves of 10‐year OS (log‐rank test) according to IHC subtype.

A total of 184 FFPE tumor samples from 176 patients with advanced breast cancer (Fig. 1A) were obtained from bone (18%), brain (12%), breast (13%, including 19 local recurrences and 5 cases of *de novo* metastatic breast cancer), liver (17%), lung (7%), lymph nodes (9%), muscle (1%), ovary (2%), peritoneum (1%), pleura (5%), and skin (15%). Clinicopathological information for all patients included is summarized in Table 1. RNA expression was analyzed in all samples using the nCounter‐based breast cancer 360™ panel of 771 genes (Fig. 1B). IHC subtypes were available from 171 samples (96.1%), and their distribution was 58.5% HR+/HER2‐negative, 10.5% HER2‐positive (HER2+), and 31% TNBC. IHC subtype was not available (N/A) for 13 samples. Median OS was 63.8 months in patients with HR+/HER2‐negative disease, 35.5 months in patients with HER2+ disease, and 22.1 months in patients with TNBC (Fig. 1C).

**Table 1:** Clinicopathological characteristics.

Characteristics		*n*
Median age at diagnosis of metastasis (range)		54 (24–89)
Menopausal status	Premenopausal	68 (38.6%)
	Postmenopausal	97 (55.1%)
	Unknown	10 (5.7%)
	Male	1 (0.6%)
Type of metastasis	De novo metastasis	27 (15.3%)
	Relapsed	146 (83.0%)
	Unknown	3 (1.7%)
Total number of metastatic sites	< 3	76 (43.2%)
	≥ 3	90 (51.1%)
	Unknown	10 (5.7%)
Site of metastatic biopsy	Locoregional	50 (27.2%)
	Distant	134 (72.8%)
Metastatic spread	Bone‐only	15 (8.5%)
	Visceral	145 (82.4%)
Organ of biopsy	Bone	33 (17.9%)
	Brain	22 (12.0%)
	Breast	24 (13.0%)
	Liver	31 (16.9%)
	Lung	13 (7.1%)
	Lymph node	16 (8.7%)
	Muscle	2 (1.1%)
	Ovary	4 (2.2%)
	Peritoneum	2 (1.1%)
	Pleura	10 (5.4%)
	Skin	27 (14.7%)
IHC group of the metastatic biopsy	HR+/HER2‐	100 (54.4%)
	HER2+	18 (9.8%)
	TNBC	53 (28.8%)
	Unknown	13 (7.1%)
PAM50 molecular subtype of the metastatic biopsy	Luminal A	30 (16.3%)
	Luminal B	47 (25.5%)
	HER2‐enriched	42 (22.8%)
	Basal‐like	54 (29.4%)
	Normal‐like	11 (6.0%)
Previous (neo)adjuvant treatment	129 (73.3%)	
Median number of lines of treatment for metastatic disease (range)	3 (0–13)	
Treatments received in the metastatic setting	Endocrine therapy	111 (63.1%)
	CDK4/6 inhibitors	84 (47.7%)
	Anti‐HER2 therapies	28 (15.9%)
	Chemotherapy	125 (71.0%)
	Immunotherapy	10 (5.7%)
	Everolimus	22 (12.5%)
	PI3K inhibitors	18 (10.2%)
	AKT inhibitors	2 (1.1%)
	Bevacizumab	15 (8.5%)
	PARP inhibitors	3 (1.7%)
Radiotherapy		111 (63.1%)

**Fig. 2:**
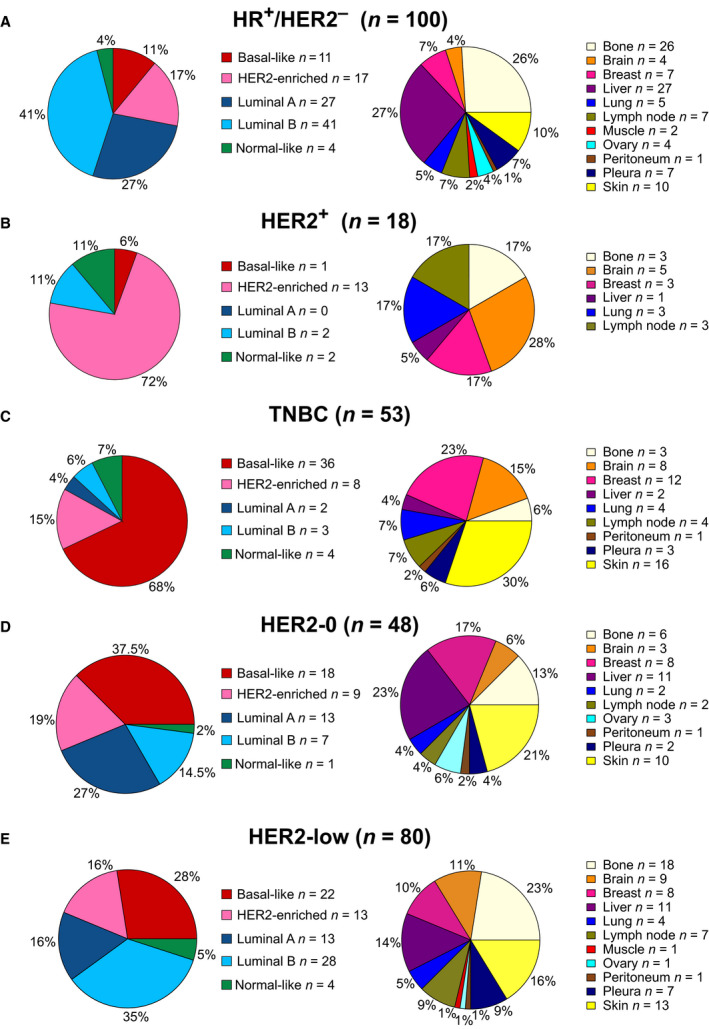
PAM50 subtype and metastatic site distribution in each IHC group. Pie charts depicting the percentage of each PAM50 subtype and the percentage of each metastatic site in (A) HR+/HER2‐negative, (B) HER2+, (C) TNBC, (D) HER2‐0, and (E) HER2‐low tumors.

The intrinsic subtype distribution was 29% basal‐like (*n* = 54), 26% luminal B (*n* = 47), 23% HER2‐enriched (*n* = 42), 16% luminal A (*n* = 30), and 6% normal‐like (*n* = 11). Within HR+/HER2‐negative disease (*n* = 100), subtype distribution was 41% luminal B (*n* = 41), 27% luminal A (*n* = 27), 17% HER2‐enriched (*n* = 17), 11% basal‐like (*n* = 11), and 4% normal‐like (*n* = 4). HR+/HER2‐negative metastatic samples were obtained from liver (27%), bone (26%), skin (10%), breast (7%), lymph nodes (7%), pleura (7%), lung (5%), brain (4%), ovary (4%), muscle (2%), and peritoneum (1%) (Fig. 2A). Within HER2+ (*n* = 18), subtype distribution was 72% HER2‐enriched (*n* = 13), 11% basal‐like (*n* = 1), 11% luminal B (*n* = 2), and 6% normal‐like (*n* = 2). HER2+ metastatic samples were obtained from bone (28%), brain (17%), breast (17%), lung (17%), lymph nodes (17%), and liver (5%) (Fig. 2B). Within TNBC (*n* = 53), subtype distribution was 68% basal‐like (*n* = 36), 15% HER2‐enriched (*n* = 8), 7% normal‐like (*n* = 4), 6% luminal B (*n* = 3), and 4% luminal A (*n* = 4). TNBC metastatic samples were obtained from skin (30%), breast (23%), brain (15%), lung (7%), lymph nodes (7%), bone (6%), pleura (6%), liver (4%), and peritoneum (2%) (Fig. 2C). Across organs, there were statistically significant differences in subtype distribution (*P* < 0.001) and IHC groups (*P* < 0.001) (Tables S1 and S2).

### 3.2 HER2‐low disease according to organ site

The HER2‐low category using IHC (i.e., HER2 1+ or HER2 2+/ISH‐negative) is becoming an important biomarker for predicting benefit from antibody–drug conjugates such as trastuzumab deruxtecan (T‐DXd) [29, 31]. However, the identification of HER2‐low disease according to organ site, with a special emphasis in bone metastasis, is unknown. To address it, we explored data of HER2 expression and *ERBB2* amplification in 146 samples (i.e., HER2‐0 *n* = 48, HER2‐low *n* = 80, and HER2+ *n* = 18). In HER2‐0 tumors (*n* = 48), the PAM50 distribution was 37.5% basal‐like (*n* = 18), 27% luminal A (*n* = 13), 19% HER2‐enriched (*n* = 9), 14.5% luminal B (*n* = 7), and 2% normal‐like (*n* = 1); HER2‐0 samples were obtained from liver (23%), skin (21%), breast (17%), bone (13%), brain (6%), ovary (6%), lung (4%), lymph nodes (4%), pleura (4%), and peritoneum (2%) (Fig. 2D). In HER2‐low metastatic tumors (*n* = 80), the PAM50 distribution was 35% luminal B (*n* = 28), 28% basal‐like (*n* = 22), 16% luminal A (*n* = 13), 16% HER2‐enriched (*n* = 13), and 5% normal‐like (*n* = 4). No significant difference in subtype distribution was identified between HER2‐0 and HER2‐low (*P* = 0.091), while a significant difference in subtype distribution was identified between HER2‐low and HER2+ (*P* < 0.001). Importantly, HER2‐low disease was identified in all metastatic sites: bone (23%), liver (14%), skin (16%), brain (11%), breast (10%), lymph nodes (9%), pleura (9%), lung (5%), muscle (1%), ovary (1%), and peritoneum (1%) (Fig. 2E). No significant difference in organ distribution was identified between HER2‐0 and HER2‐low (*P* = 0.414) nor between HER2‐low and HER2+ (*P* = 0.207).

**Fig. 3:**
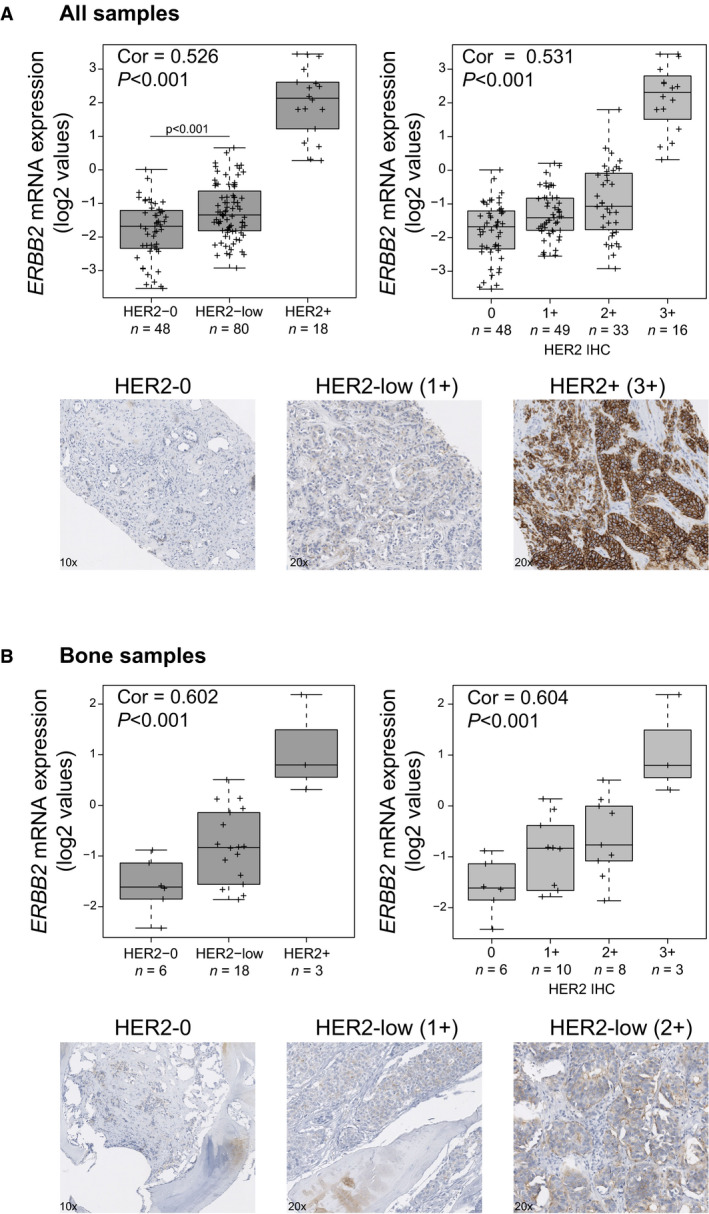
*ERBB2* mRNA correlates with HER2 protein expression. *ERBB2* mRNA expression (log2 values) across HER2 IHC categories (i.e., HER2‐0, HER2‐low, and HER2+ or HER2 0+, 1+, 2+, and 3+) in (A) all metastatic sites and (B) bone metastasis. Spearman correlation was determined between *ERBB2* mRNA and HER2 protein expression. Examples of HER2 staining are represented at 10× and 20×.

Technical aspects might affect IHC staining of metastatic lesions, including bone metastasis. To address this potential issue, we correlated the expression of HER2 protein levels with *ERBB2* mRNA and the expression of ER protein levels with *ESR1* mRNA. Overall, *ERBB2* mRNA was found significantly correlated with HER2 protein levels (HER2‐0, HER2‐low, or HER2+) (Spearman Cor = 0.531, *P* < 0.001). Compared to HER2‐0 disease, *ERBB2* mRNA levels were found increased 1.20‐fold, 4.44‐fold, and 14.04‐fold in HER2 1+, HER2 2+/ISH‐negative, and HER2+ disease, respectively (Fig. 3A). In bone metastases, which are usually not accepted for inclusion in clinical trials, *ERBB2* mRNA levels were also significantly correlated with HER2 protein levels (Spearman Cor = 0.604, *P* < 0.001) (Fig. 3B). We also found significant correlation between *ERBB2* mRNA and HER2 protein levels in brain metastasis (Spearman Cor = 0.697, *P* = 0.002), breast metastasis (Spearman Cor = 0.754, *P* = 0.002), lung metastasis (Cor = 0.882, *P* = 0.003), and lymph node metastasis (Spearman Cor = 0.410, *P* = 0.05). Importantly, a strong correlation between ER protein expression determined by IHC and *ESR1* mRNA was observed across all metastatic samples (Pearson Cor = 0.82, *P* < 0.001) and across bone metastatic samples (Pearson Cor = 0.85, *P* < 0.001) (Fig. S1), suggesting that good quality gene expression data can be obtained from bone samples.

### 3.3 Effect of organ site in gene expression profiling

**Fig. 4:**
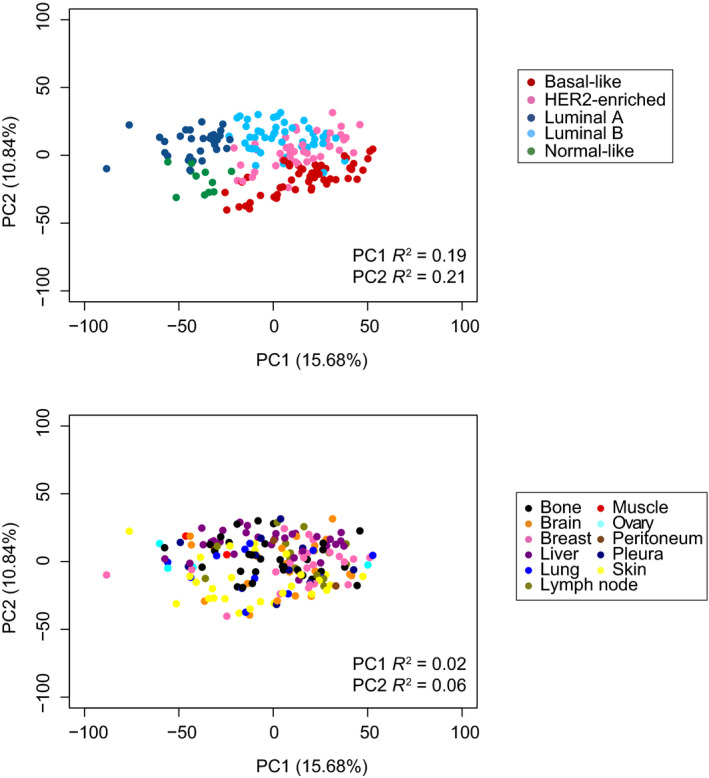
Principal component analysis. Unsupervised PCA of 181 metastatic samples with coloring of PAM50 molecular subtype and metastatic site. % of gene expression variability explained by each PC, and PC1 and PC2 R^2^ values obtained from simple linear regression models are show.

The influence of organ site in gene expression profiling of metastatic breast cancer has not been formally addressed. To start approaching it, we first assessed the expression of 771 breast cancer‐related genes across the 184 metastatic tumor samples and 11 organ sites. Three out of 184 samples were identified as outliers and were excluded from the gene expression analysis (Fig S2). Unsupervised hierarchical clustering (Fig. S3) and principal component analysis (Fig. 4) revealed that the intrinsic subtypes explain a greater amount of gene expression variability than organ of metastasis. Secondly, we combined gene expression data from 186 patients with early‐stage breast cancer representative of all subtypes in the 181 metastatic dataset. The combined dataset (*n* = 367) of tumors obtained from early‐stage and metastatic breast cancer revealed that the 2 main principal components (i.e., PC1 and PC2) are also explained by intrinsic subtype (Fig. S4).

### 3.4 Genes and biological processes associated with organ site

To explore differences in gene expression across metastatic sites, we performed a multiclass SAM analysis. Using a FDR < 5%, we identified a total of 631 differentially expressed genes (81.1%) across organs (Table S3). Some examples were *IBSP,* which was highly expressed in bone metastasis; *FGF1,* which was highly expressed in brain metastasis; *PCK1,* which was highly expressed in liver metastasis; *CAV1,* which was highly expressed in lung metastasis; or *KRT14,* which was highly expressed in skin metastasis. Next, we performed a two‐class unpaired SAM analysis between each organ versus the rest of samples to identify genes whose expression is associated with each organ of metastasis. Using a FDR < 5%, we identified a total of 518 upregulated genes (67.2%) across organs (i.e., 204 in bone, 201 in skin, 109 in brain, 91 in liver, 29 in lung, and 7 in breast) (Table S4 and Fig. S5A). We did not identify any upregulated gene in lymph node or pleural metastases, nor any common gene upregulated in all metastatic sites (Fig. S5B). We also compared the bone samples of patients with bone‐only metastasis vs patients with metastasis in bone and other sites, and we could not find any significant differential expressed gene (Table S4).

We then carried out functional enrichment GO analyses using the upregulated gene lists, and although these analyses were limited by a minority of genes in each gene list, they revealed biological processes and pathways significantly enriched (*P* < 0.05) in each metastatic site (Table S5). In bone metastasis, ossification, the bone morphogenetic protein (BMP), the TGF‐beta, and the Hippo signaling pathways were enriched. In brain metastases, enriched GO and pathways included regulation of transcription and GTPase activity and cell migration and also brain‐related processes such as nervous system development, chemical synaptic transmission, adult behavior, dopamine synapse, or amphetamine addiction. In liver metastases, we identified enrichment in processes such as oxidation–reduction, glucose metabolism, chromatin remodeling, cholesterol esterification or vasodilation, and the AMPK and calcium signaling pathway. In lung metastasis, enriched GO and pathways included regulation of transcription, immune response, regulation of nitric oxide regulation, regulation of IL6 production, or regulation of vasoconstriction. Finally, enriched GO and pathways in skin metastasis included cell adhesion, angiogenesis, extracellular matrix organization, proteolysis, wound healing, epidermis development, collagen‐related processes and ERK and Notch pathways and lipid metabolism (Fig. S6).

The previous gene expression results could be confounded by differences in subtype distribution across organs. To identify genes whose high expression was specific of metastatic site and independent of subtype, we performed adjusted logistic regression analysis for each individual gene. A total of 74 genes were identified (*P* < 0.05): 36 bone‐specific genes, 18 liver‐specific genes, 12 brain‐specific genes, and 8 skin‐specific genes (Table 2). Of note, we identified known organ‐specific genes such as the integrin‐binding sialoprotein (*IBSP*) for bone, the crystallin alpha B (*CRYAB*) for brain, the aldehyde dehydrogenase 1 family member A1 (*ALDH1A1*) for liver, or *KRT14* for skin. In addition, we identified 3 genes found in the PAM50 gene list to be associated with bone (*FOXC1)* and skin (*KRT14* and *KRT5*) metastasis.

**Table 2:** Subtype‐independent organ‐specific genes.

Gene	Gene description	Gene location	Metastatic site	*P*‐value	Lawler *et al*.
*WIF1*	WNT Inhibitory Factor 1	12q14.3	Bone	8.94E‐07	High expression in primary tumors associated with bone+visceral metastasis
*IBSP*	Integrin‐binding Sialoprotein	4q22.1	Bone	1.37E‐06	
*MMP9*	Matrix Metallopeptidase	20q13.12	Bone	2.44E‐06	
*ITGB3*	Integrin Subunit Beta 3	17q21.32	Bone	2.74E‐06	
*VIT*	Vitrin	2p22.2	Bone	3.25E‐06	High expression in primary tumors associated with bone+visceral metastasis
*HBB*	Hemoglobin Subunit Beta	11p15.4	Bone	1.40E‐05	
*WNT5B*	Wnt Family Member 5B	12p13.33	Bone	3.01E‐05	High expression in primary tumors associated with visceral‐only
*CHAD*	Chondroadherin	17q21.33	Bone	3.22E‐05	High expression in primary tumors associated with bone‐only
*BMP2*	Bone Morphogenetic Protein 2	20p12.3	Bone	3.38E‐05	
*EYA1*	EYA Transcriptional Coactivator And Phosphatase 1	8q13.3	Bone	5.05E‐05	High expression in primary tumors associated with bone‐only
*FOXC2*	Forkhead Box C2	16q24.1	bone	8.48E‐05	High expression in primary tumors associated with bone+visceral metastasis
*FZD8*	Frizzled Class Receptor 8	10p11.21	bone	0.0001	
*OLFML2B*	Olfactomedin‐like 2B	1q23.3	bone	0.0001	
*TGFB1*	Transforming Growth Factor Beta 1	19q13.2	bone	0.0004	High expression in primary tumors associated with bone‐only
*BMP5*	Bone Morphogenetic Protein 5	6p12.1	bone	0.0005	
*ENPP2*	Ectonucleotide Pyrophosphatase/Phosphodiesterase 2	8q24.12	bone	0.0006	High expression in primary tumors associated with visceral‐only
*NUDT1*	Nudix Hydrolase 1	7p22.3	bone	0.0014	High expression in primary tumors associated with visceral‐only
*FGF7*	Fibroblast Growth Factor 7	15q21.2	bone	0.0015	
*FOXC1*	Forkhead Box C1	6p25.3	bone	0.0024	High expression in primary tumors associated with visceral‐only
*BMP8A*	Bone Morphogenetic Protein 8a	1p34.3	bone	0.0044	
*EYA4*	EYA Transcriptional Coactivator And Phosphatase 4	6q23.2	bone	0.0045	
*RNASE2*	Ribonuclease A Family Member 2	14q11.2	bone	0.006	
*SRPX*	Sushi‐repeat Containing Protein X‐linked	Xp11.4	bone	0.006	
*MME*	Membrane Metalloendopeptidase	3q25.2	bone	0.0143	High expression in primary tumors associated with bone+visceral metastasis
*LIFR*	LIF Receptor Subunit Alpha	5p13.1	bone	0.0146	
*BAX*	BCL2‐associated X, Apoptosis Regulator	19q13.33	bone	0.0192	High expression in primary tumors associated with bone‐only
*SCARA5*	Scavenger Receptor Class A Member 5	8p21.1	bone	0.0211	
*EYA2*	EYA Transcriptional Coactivator And Phosphatase 2	20q13.12	bone	0.0219	High expression in primary tumors associated with visceral‐only
*XRCC3*	X‐ray Repair Cross‐complementing 3	14q32.33	bone	0.0268	
*LEPR*	Leptin Receptor	1p31.3	bone	0.0281	
*BCL2L1*	BCL2 Like 1	20q11.21	bone	0.0325	
*NCAM1*	Neural Cell Adhesion Molecule 1	11q23.2	bone	0.0342	
*SMAD3*	SMAD Family Member 3	15q22.33	bone	0.0368	
*RAC2*	Rac Family Small GTPase 2	22q13.1	bone	0.0449	High expression in primary tumors associated with visceral‐only
*HOXA9*	Homeobox A9	7p15.2	bone	0.0483	High expression in primary tumors associated with bone‐only
*CKB*	Creatine Kinase B	14q32.33	bone	0.049	High expression in primary tumors associated with visceral‐only
*CRYAB*	Crystallin Alpha B	11q23.1	brain	0.0006	
*NRCAM*	Neuronal Cell Adhesion Molecule	7q31.1	brain	0.0007	
*FGF1*	Fibroblast Growth Factor 1	5q31.3	brain	0.0008	High expression in primary tumors associated with bone+visceral metastasis
*GDF15*	Growth Differentiation Factor 15	19p13.11	brain	0.0021	
*SOX2*	SRY‐Box Transcription Factor 2	3q26.33	brain	0.0049	High expression in primary tumors associated with bone+visceral metastasis
*GRIN1*	Glutamate Ionotropic Receptor NMDA Type Subunit 1	9q34.3	brain	0.0075	
*RASGRF1*	Ras Protein‐specific Guanine Nucleotide‐releasing Factor 1	15q25.1	brain	0.0103	High expression in primary tumors associated with visceral‐only
*SOX10*	SRY‐Box Transcription Factor 10	22q13.1	brain	0.0199	
*CHI3L1*	Chitinase 3‐like 1	1q32.1	brain	0.0223	High expression in primary tumors associated with visceral‐only
*ZIC2*	Zic Family Member 2	13q32.3	brain	0.0276	
*NRXN1*	Neurexin 1	2p16.3	brain	0.0447	
*LEFTY2*	Left–Right Determination Factor 2	1q42.12	brain	0.0495	
*ALDH1A1*	Aldehyde Dehydrogenase 1 Family Member A1	9q21.13	liver	5.55E‐05	
*CYP4F3*	Cytochrome P450 Family 4 Subfamily F Member 3	19p13.12	liver	5.67E‐05	High expression in primary tumors associated with bone‐only
*PCK1*	Phosphoenolpyruvate Carboxykinase 1	20q13.31	liver	7.46E‐05	
*RELN*	Reelin	7q22.1	liver	0.0002	
*AGT*	Angiotensinogen	1q42.2	liver	0.0004	
*PPARGC1A*	PPARG Coactivator 1 Alpha	4p15.2	liver	0.0004	
*HNF1A*	HNF1 Homeobox A	12q24.31	liver	0.0009	
*CDH2*	Cadherin 2	18q12.1	liver	0.0029	
*APOE*	Apolipoprotein E	19q13.32	liver	0.0053	
*GGH*	Gamma‐Glutamyl Hydrolase	8q12.3	liver	0.0082	High expression in primary tumors associated with visceral‐only
*HGF*	Hepatocyte Growth Factor	7q21.11	liver	0.0159	
*MT1G*	Metallothionein 1G	16q13	liver	0.016	
*CLDN1*	Claudin 1	3q28	liver	0.017	
*UBB*	Ubiquitin B	17p11.2	liver	0.0173	
*HDAC1*	Histone Deacetylase 1	1p35.2‐p35.1	liver	0.0207	
*EDNRB*	Endothelin Receptor Type B	13q22.3	liver	0.0292	
*GATA4*	GATA‐binding Protein 4	8p23.1	liver	0.0444	
*MARCO*	Macrophage Receptor With Collagenous Structure	2q14.2	liver	0.0489	High expression in primary tumors associated with visceral‐only
*KRT14*	Keratin 14	17q21.2	skin	0.0005	High expression in primary tumors associated with bone+visceral metastasis
*KRT5*	Keratin 5	12q13.13	skin	0.0029	
*S100A7*	S100 Calcium‐binding Protein A7	1q21.3	skin	0.0044	
*SERPINB5*	Serpin Family B Member 5	18q21.33	skin	0.0069	High expression in primary tumors associated with bone+visceral metastasis
*MMP3*	Matrix Metallopeptidase 3	11q22.2	skin	0.0116	
*IL20RB*	Interleukin 20 Receptor Subunit Beta	3q22.3	skin	0.0133	High expression in primary tumors associated with visceral‐only
*SFN*	Stratifin	1p36.11	skin	0.021	
*TPSAB1*	Tryptase Alpha/Beta 1	16p13.3	skin	0.0333	

Finally, we interrogated the 74 genes in 390 breast primary tumors from Lawler *et al*. [23] publicly available dataset, where 3 types of metastatic spread have been identified: bone and visceral metasynchronous spread, bone‐only spread, and visceral‐only metastasis. Among the 74 genes, 26 genes (35.1%) were found significantly associated with the type of metastatic spread, including 5 bone‐specific genes (*CHAD, EYA1, TGFB1, BAX,* and *HOXA9*) whose high expression was associated with bone‐only metastasis, 4 bone‐specific genes (*WIF1, VIT, FOXC2,* and *MME*) whose high expression was associated with bone and visceral metastasis, 2 brain‐specific genes (*FGF1* and *SOX2*) whose high expression was associated with bone and visceral metastasis, 2 brain‐specific genes (*RASGRF1* and *CHI3L1*) whose high expression was associated with visceral‐only metastasis, and 2 liver‐specific genes (*GGH* and *MARCO*) whose high expression was associated with visceral‐only metastasis (Table 2). This result suggests that particular metastatic organ‐specific genes might also be indicative of the type of metastatic spread when analyzed in primary tumors.

### 3.5 Immune expression profiles across organ sites

**Fig. 5:**
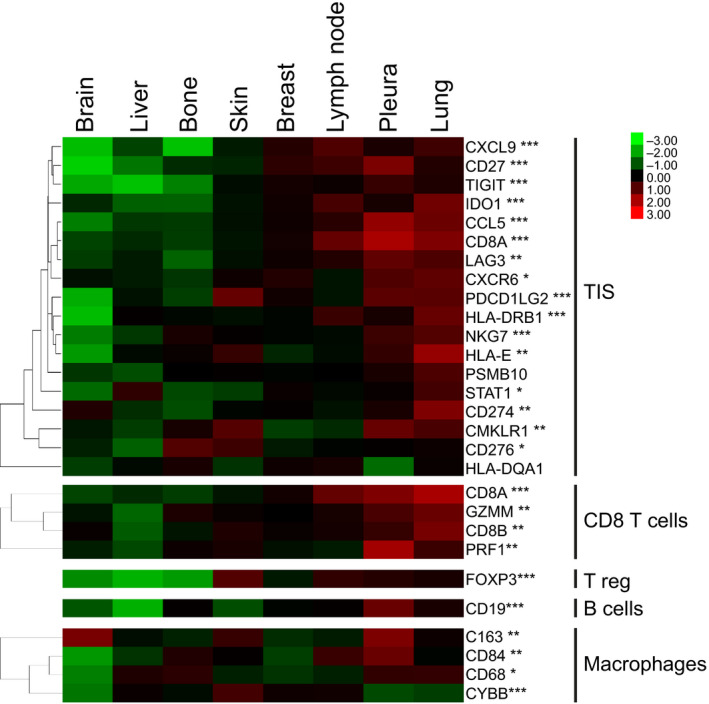
Differential expression of immune genes across metastatic sites. Expression of genes comprised in immune signatures across metastatic sites. The heatmap shows high (red) to low (green) expression of mRNAs in each metastatic site. Significant changes across metastatic sites by multiclass SAM analysis are indicated: *FDR < 5%, **FDR < 1%, and ***FDR < 0.1%.

We then investigated differences in the expression of 95 immune genes across metastatic sites. The expression of 89 genes was found significantly different across metastatic sites (FDR < 5%) (Table S6 and Fig. S7A). Among them, we identified 18 genes of the tumor inflammation signature (TIS) (*CCL5, CD27, CD276, CD274, CD8A, CMKLR1, CXCL9, CXCR6, HLA‐DQA1, HLA‐DRB1, HLA‐E, IDO1, LAG3, NKG7, PDCD1LG2, PSMB10, STAT1,* and *TIGIT*) which has been previously associated with anti‐PD‐1/PD‐L1 response [28,29], 4 genes associated with CD8 T cells (*CD8A, GZMM, CD8B,* and *PRF1*), a marker of functional regulatory T cells (Treg) (*FOXP3*), a biomarker for B cells (*CD19*), and 4 macrophage‐related genes (*C163, CD84, CD68, and CYBB*) (Fig. 5). Lung and pleura were the sites with higher expression of immune genes, while brain had the lowest expression of immune genes. Moreover, we found 86 immune genes differentially expressed across the molecular subtypes (FDR < 5%) (Table S7). Basal‐like was the subtype with the highest expression of immune genes (Fig. S7B). Finally, to identify immune genes whose expression was specific of metastatic site and independent of subtype, we performed adjusted logistic regression analysis for each individual immune gene. Regardless of molecular subtype, we identified 27 highly expressed genes in lung, 23 highly expressed genes in pleura, 18 highly expressed and 9 lowly expressed genes in bone, 10 highly expressed and 21 lowly expressed genes in liver, or 7 highly expressed and 39 lowly expressed genes in brain (Table S8).

### 3.6 Associations with overall survival

**Fig. 6:**
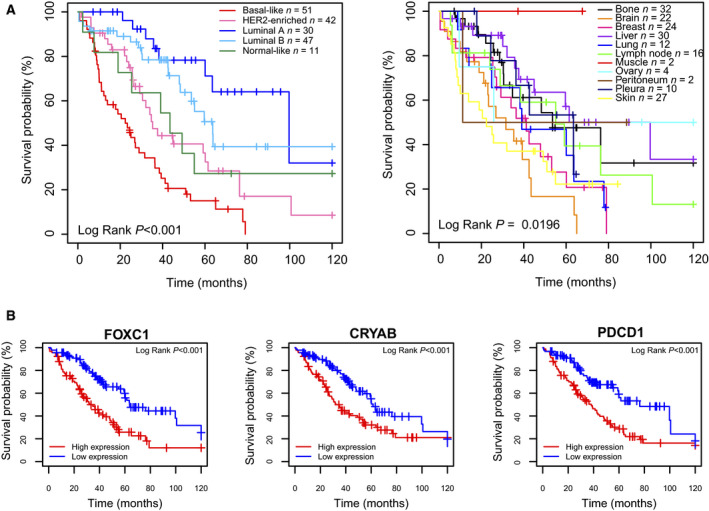
Associations with overall survival. (A) Kaplan–Meier curves of 10‐year OS (log‐rank test) according to PAM50 molecular subtype and metastatic site. (B) Examples genes associated with poor OS, including two organ‐specific genes (*FOXC1* and *CRYAB*) and the immune gene *PDCD1*. Kaplan–Meier curves of 10‐year OS (log‐rank test) according to median gene expression of the selected genes.

We evaluated the prognostic ability of the PAM50 subtypes and the site of metastasis. PAM50 molecular subtypes were associated with OS (*P* < 0.001) and better discriminated prognosis than site of metastasis (Fig. 6A). Median OS was 99.7 months for luminal A, 63.6 for luminal B, 34.7 months for HER2‐enriched, and 22.4 months for basal‐like.

We then explored the association of 771 individual genes and 9 signatures with OS. We identified 1 signature score (i.e., luminal A signature score) and 51 genes whose high expression was significantly associated with better OS, and 2 signature scores (i.e., basal‐like signature score and PAM50MET signature score [27]) and 25 genes whose high expression was significantly associated with worse OS (Table S9). When adjusting for PAM50 subtype, 45 of the 771 genes (5.8%) were significantly associated with OS (Fig. S8 and Table S10), of which the high expression of 9 genes was associated with worse OS (*ENO1, CDCA5, FAM83D, ANLN, MMP7, CRYAB, FOXC1, E2F1,* and *PDCD1*) including the bone‐specific gene *FOXC1*, the brain‐specific genes *CRYAB,* or the immune‐related gene *PDCD1* (Fig. 6B). Finally, we performed a multivariate analysis adjusting for clinicopathological variables including menopausal status, type of metastasis (de novo or relapsed), number of metastatic sites, metastatic site of the biopsy, PAM50 molecular subtype, and number of lines of therapy and found that 5 of the 9 genes were still significantly associated with worse OS (*FAM83D, ANLN, CRYAB, FOXC1,* and *E2F1*) (Table S10).

## 4 Discussion

To our knowledge, this is the first study to evaluate gene expression profiles of breast cancer across metastatic organs. In particular, we explored genomic differences between sites of metastatic disease and made the following observations: (a) All intrinsic molecular subtypes are identified within IHC groups; (b) HER2‐low disease is identified in all metastatic sites; (c) intrinsic molecular subtypes determined in the metastatic site are associated with OS regardless of where biopsy was performed; (d) lung and pleural metastases have the highest expression of immune genes, while brain and liver have the lowest; and (e) the expression of individual genes is organ‐specific and is associated with OS.

Previously, we reported that approximately 15% of primary luminal A and B HR+/HER2‐negative tumors become HER2‐enriched once they metastasize, regardless of HER2 status [11, 17]. Concordant with this observation, here we observed a higher frequency of HER2‐enriched and basal‐like subtypes in HR+/HER2‐negative metastasis compared to primary tumors [17]. On the other side, in primary HER2+ disease we have previously reported 47% of HER2‐enriched, 24% of luminal A, 20% of luminal B, and 9% of basal‐like tumors. Here, despite the small HER2+ metastatic sample size, we did not detect luminal A tumors, while 70% were HER2‐enriched. Finally, approximately 60–80% of TNBC primary tumors have been reported to be basal‐like and 9% HER2‐enriched [32, 33] and our results showed similar distribution of molecular subtypes in metastatic TNBC.

The acquisition of more aggressive molecular subtypes in the metastatic setting, such as HER2‐enriched and basal‐like [34], especially in HR+/HER2‐negative disease, may be due to patient selection, changes in the tumor biology due to its inherent evolution, the effects of therapies, or a combination of all. Recently, comprehensive genomic studies of metastatic breast cancers linked an increase in APOBEC genetic signatures with metastatic HR+/HER2‐negative breast cancer [14, 35]. Interestingly, high frequency of APOBEC3B‐associated mutations occurs in HER2‐enriched subtype [36] which is consistent with the increase in this subtype observed in the metastatic setting.

Here, we also report HER2‐low disease in all metastatic sites, including bone. Bone metastases are usually not accepted for inclusion in clinical trials due to decalcification procedures related to IHC. Here, we show that *ERBB2* mRNA is highly correlated with HER2 IHC in bone metastasis, suggesting that bone metastasis might be a reliable organ to detect HER2 expression. Nonetheless, alternative quantitative measurements of HER2 (i.e., *ERBB2* mRNA) may help better identify patients who might benefit from potent anti‐HER2 antibody–drug conjugates, like T‐DXd [31, 37].

Interestingly, we have previously observed in a wide population of almost 1600 HER2‐negative tumors that HER2‐low disease was enriched in luminal molecular subtypes (about 80%), especially when compared to HER2‐0 (about 50%) [29]. In the present study on a smaller sample size (80 HER2‐low specimens), luminal subtypes accounted for roughly half of the total and no difference in subtypes distribution was observed between HER2‐low and HER2‐0 tumors. However, in our previous study, only 2.4% of HER2‐low tumors were metastatic. A potential shift in molecular subtype distribution between primary and metastatic tumors might thus merit a more careful evaluation, along with its potential prognostic and therapeutic implications.

Our study identified particular genes differentially expressed across metastatic sites, suggesting a potential role of the tumor microenvironment. Indeed, our functional enrichment analysis of upregulated genes in each metastatic site identified biological processes and pathways related with the organ where metastasis was seeded. Moreover, we have validated some previously reported overexpressed genes such as *TGFB1, IBSP, MMP9,* or *ITGB3* in bone metastasis [4, 38, 39]; *CRYAB, NRCAM,* and *SOX2* in brain metastasis [40, 41, 42]; *VEGF* and *IL6* in lung metastasis [4, 43]; and *CYP4F3* in liver metastasis [44]. *ALDH1A1, PCK1,* and *APOE* were previously described to be upregulated in liver metastases of colorectal cancer [45, 46, 47]. Further studies are required to understand whether these genes could be used as therapeutic targets or biomarkers of response.

Our data indicate that lung and pleura are the sites of metastasis with higher expression of immune genes, while brain and liver have the lowest expression of immune genes. This is consistent with the findings of a recently published study of over 400 metastatic samples which indicate that lung metastasis has the highest TIS compared to other metastatic sites regardless of the cancer of origin [48]. Notably, high TIS is a biomarker of response to immunotherapy [49, 50]. Taken together, these data suggest that patients with lung and pleural metastasis might benefit from immune checkpoint blockade, while other treatment approaches could be more suitable for liver and brain metastases.

Concordant with early‐stage breast cancer [51], PAM50 subtypes in metastatic tissues were found highly prognostic. At the same time, we identified 45 genes whose expression provides prognostic information beyond PAM50 subtypes. For example, we identified high PD1 expression as being associated with poor prognosis. Functional studies are needed to better understand whether the genes associated with worse OS could also be therapeutic targets. Indeed, high PD1 mRNA might be a tumor‐agnostic biomarker of benefit from anti‐PD1 therapy [52].

Our study has some limitations worth noting. First, this is a retrospective study using the available metastatic tumor samples at Hospital Clinic of Barcelona and a set of 23 TNBC samples from Hospital Universitario 12 de Octubre; therefore, selection bias is likely. For instance, we had a very small HER2+ sample size. However, the distribution of IHC groups was very similar to the seminal work by Bertucci and colleagues [14]. On the other hand, the distribution of the organs of the selected biopsies may not reflect the actual frequency of breast cancer metastatic sites due to the accessibility to the organs of metastasis. Second, our cohort is very heterogeneous in terms of systemic therapies received. Thus, we could not link the biological findings with treatment benefit. Third, our dataset surprisingly had longer median OS than expected, possibly because those patients that have biopsies are the more likely to survive longer. Indeed, having a biopsy upon recurrence has been associated with longer survival [53]. Fourth, our analyses are limited to 771 genes; whether different results might be obtained with more genes is unknown. Fifth, we did not explore the biological differences across metastatic sites within a single patient.

## 5 Conclusion

In summary, although main molecular features from primary tumors are known to be maintained in advanced disease [9, 10], here we report higher proportion of aggressive molecular subtypes in the metastatic setting, especially in HR+/HER2‐negative disease, and unique biological features of each metastatic site, indicating a role of the tumor microenvironment and the need to biopsy metastatic disease in patients with advanced breast cancer to better select the treatment strategy for each patient. Understanding the biology of each metastatic site can potentially impact the design of new therapies and ultimately improve patient outcomes. Finally, our study provides a precious dataset of cancer metastasis that can be further exploited.

## Conflict of interest

Potential conflicts of interest are the following: A.P. reports advisory and consulting fees from Roche, Pfizer, Novartis, Amgen, BMS, Puma, Oncolytics Biotech, MSD, Guardant Health, Peptomyc, and Lilly, lecture fees from Roche, Pfizer, Novartis, Amgen, BMS, NanoString Technologies, and Daiichi Sankyo, institutional financial interests from Boehringer, Novartis, Roche, NanoString, Sysmex Europe GmbH, Medica Scientia inno. Research, SL, Celgene, Astellas, and Pfizer; a leadership role in Reveal Genomics, SL; and a patent PCT/EP2016/080056.

## Author contributions

FB‐M and AP performed experimental study design. FB‐M, NC, OM‐S, TP, MM‐L, BG‐F, ES, DM, PG, EB, PT, ECi, ECa, BA, RM, MV, MM, and AP acquired the data. FB‐M, LP, FS, BG‐F, ES, DM, PG, EB, and AP analyzed the data. FB‐M, NC, OM‐S, TP, MM‐L, BG‐F, ES, PT, ECi, MM, and AP interpreted the data. FB‐M and AP wrote the manuscript. All authors reviewed the manuscript.

### Peer Review

The peer review history for this article is available at https://publons.com/publon/10.1002/1878‐0261.13021.

## Supporting information


**Fig. S1**. Correlation between ESR1 mRNA and % ER protein expression. Pearson correlation between ESR1 mRNA and ER protein expression across all metastatic sites (n = 148) and across bone metastasis (n = 29) with coloring of PAM50 molecular subtype.

**Fig. S2**. PCA in 184 metastatic tumors. Unsupervised PCA of 181 metastatic tumors with coloring of samples included in the gene expression analyses (grey) and the outliers, excluded from gene expression analyses (red).

**Fig. S3**. Gene expression features. Unsupervised hierarchical clustering of 181 metastatic samples. Heatmaps show high (red) to low (green) expression of mRNAs in each sample. The organ of biopsy, IHC and PAM50 molecular subtype of each sample are shown.

**Fig. S4**. PCA in primary and metastatic tumors. Unsupervised PCA of 186 primary and 181 metastatic tumors with coloring of type of biopsy (primary [P] vs metastatic [M]) and PAM50 molecular subtypes.

**Fig. S5**. Differential gene expression across metastatic sites. (A) Volcano plots showing differentially expressed genes in each organ vs others (B) Venn diagram showing common significantly upregulated genes in all metastatic sites. Each circle includes the number of genes upregulated and the sites where these genes are upregulated.

**Fig. S6**. Functional enrichement analysis of upregulated genes. Gene ontology (GO) and KEGG pathway analysis were performed using DAVID. Significantly enriched (p < 0.05) biological processes and pathways are presented. Functional enrichement analysis of upregulated genes. Gene ontology (GO) and KEGG pathway analysis were performed using DAVID. Significantly enriched (p < 0.05) biological processes and pathways are presented.

**Fig. S7**. Expression of immune genes. Differential expression of immune genes across (A) metastatic sites and (B) PAM50 molecular subtypes. Heatmaps show high (red) to low (green) expression of RNAs in each sample. Significantly different gene expression were identified using multiclass SAM (*FDR<5%, **FDR<1%, ***FDR<0.01%).

**Fig. S8**. Associations with overall survival. Forest plot showing genes and signatures associated with OS.

**Table S1**. Distribution of PAM50 subtypes and IHC groups across metastatic sites.
**Table S2**. Distribution of PAM50 subtypes in each metastatic site according to IHC group.
**Table S3**. Multiclass SAM of 771 genes between metastatic sites.
**Table S4**. Unpaired SAM between each metastatic site and others.
**Table S5**. Functional enrichment GO analyses of the up‐regulated genes in each metastatic site.
**Table S6**. Multiclass SAM of 95 immune genes between metastatic sites.
**Table S7**. Multiclass SAM of 95 immune genes between molecular subtypes.
**Table S8**. Adjusted logistic regression analysis for 95 immune genes.
**Table S9**. Univariate analysis to investigate the association of 771 individual genes and 9 signatures with OS.
**Table S10**. Bivariate analysis (adjusted for PAM50) and multivariate analysis (adjusted for menopausal status, type of metastasis, number of metastatic sites, metastatic site of the biopsy, PAM50 subtype, number of lines of therapy) to investigate the association of 771 individual genes and 9 signatures with OS.

## Data Availability

The data that support this study are available in Tables S1–10 and are derived from the gene expression data deposited in the Gene Expression Omnibus under the accession number GSE175692.
